# From Cycling Between Coupled Reactions to the Cross-Bridge Cycle: Mechanical Power Output as an Integral Part of Energy Metabolism

**DOI:** 10.3390/metabo2040667

**Published:** 2012-10-08

**Authors:** Frank Diederichs

**Affiliations:** Marschweg 10, D-29690 Schwarmstedt, Germany; Email: fwkh.diederichs@googlemail.com

**Keywords:** energetic coupling, zero resistance, cross-bridge cycle, muscular efficiency, muscular fatigue

## Abstract

ATP delivery and its usage are achieved by cycling of respective intermediates through interconnected coupled reactions. At steady state, cycling between coupled reactions always occurs at zero resistance of the whole cycle without dissipation of free energy. The cross-bridge cycle can also be described by a system of coupled reactions: one energising reaction, which energises myosin heads by coupled ATP splitting, and one de-energising reaction, which transduces free energy from myosin heads to coupled actin movement. The whole cycle of myosin heads via cross-bridge formation and dissociation proceeds at zero resistance. Dissipation of free energy from coupled reactions occurs whenever the input potential overcomes the counteracting output potential. In addition, dissipation is produced by uncoupling. This is brought about by a load dependent shortening of the cross-bridge stroke to zero, which allows isometric force generation without mechanical power output. The occurrence of maximal efficiency is caused by uncoupling. Under coupled conditions, Hill’s equation (velocity as a function of load) is fulfilled. In addition, force and shortening velocity both depend on [Ca^2+^]. Muscular fatigue is triggered when ATP consumption overcomes ATP delivery. As a result, the substrate of the cycle, [MgATP^2−^], is reduced. This leads to a switch off of cycling and ATP consumption, so that a recovery of [ATP] is possible. In this way a potentially harmful, persistent low energy state of the cell can be avoided.

## 1. Introduction

In cellular metabolism, energy transductions are brought about by coupled reactions. The network of energy metabolism is organised in such a way that cycling of respective intermediates, like protons in oxidative phosphorylation (OP), or [Pi], [ADP], and [ATP] in the ATP cycle, is ensured. As was shown previously [1], entropy production during steady state cycling must be zero. This follows from the fact that the line integral taken around a closed path is zero if the integrand is an exact differential. This latter constraint is always fulfilled for potential functions like electro-chemical potentials or affinities. It will be shown that this steady state cycling between coupled reactions is associated with the occurrence of negative resistances. 

In a muscle fiber, mechanical power output is coupled to ATP splitting. How this is achieved is not fully understood, although there has been great success in many field endeavours in muscular research such as the structure of the contractile apparatus and its functional correlates [2,3,4,5,6]. Since Huxley’s widely accepted sliding filament theory [7,8], the cross-bridge cycle is of central importance, especially in the functional aspects of contraction. This cycle must contain the reactions of free energy transduction from chemical (ATP splitting reaction) to mechanical energy (actin movement against a load force). From the overall reaction, contractile efficiency can be obtained by relating mechanical power output to the dissipation function of ATP splitting, where the mechanical power is given by the product of the force exerted by the load and the shortening velocity. Experimental results show [9,10,11,12], that when efficiency is expressed as a function of *v*, a curved line with a maximum is obtained. From non-equilibrium thermodynamics (NET, [13]), it is well known that uncoupling is necessary to generate a maximum in efficiency plots (efficiency against reduced force ratio). Thus, to yield such a maximal efficiency, any description of the cross-bridge cycle on a thermodynamic basis must contain an uncoupling mechanism, which uncouples the transduction of free energy from ATP splitting to actin movement. 

To describe the cross-bridge cycle in terms of the new flux equations published recently [1], the cross-bridge cycle has to be formulated in relation to this formalism, which combines the basics of NET [13,14,15,16] with Michaelis-Menten-like kinetics of enzyme-catalysed reactions [17]. It will be shown that Hill’s equation describing muscular performance [18,19] can be easily deduced by applying the new flux equation. 

When compared with other approaches to the energetics of the cross-bridge cycle, the main particularity of the present work may be the fact that this cycle is connected here to energy metabolism of the muscle fiber, *i.e.*, to ATP producing and consuming reactions. The generation of mechanical energy from the free energy of ATP splitting is treated here as one of the parallel reactions of the sarcosol consuming ATP delivered in fast fibers, mainly from glycogenolysis or glycolysis, respectively. This integration into the cell’s energy metabolism makes it possible to inspect some variables like ATP and its reaction products and species at high mechanical power output. In addition, concentration changes in metabolites and ions like creatine phosphate, lactate, H^+^, and Mg^2+^, are of interest under these conditions. This is achieved by formulating, in particular, the ATP splitting reaction according to Alberty [20] as a function of both [H^+^] and [Mg^2+^]. 

It is the aim of this study to elucidate cycling between coupled reactions, and if such cycling is also involved with the cross-bridge cycle and force generation. In addition, the consequences of uncoupling on power output and efficiency will be shown. For this, a formulation of the cycle in terms of the above mentioned new flux equation had to be derived. 

The phenomenon of muscular fatigue at the cellular level occurs when ATP consumption exceeds ATP delivery [21,22,23,24,25]. Under such conditions drastic changes in many metabolite and ion concentrations can be expected. The results of simulations will show to what extent these changes may contribute to fatigue, and if this phenomenon can be explained by such changes alone. 

## 2. Results and Discussion

### 2.1. How Negative Conductances Are Generated

In a previous article [1] it was shown that at steady state all affinities and dissipation functions of closed pathways associated with coupled in series reactions must vanish. In the following, it will be demonstrated that the overall resistance (=1/conductance) of such cycles must also be zero. As a consequence, the existence of negative conductances (or resistances) has to be called for. 

According to [1] a coupled two-flux-system can be described as: 

*J_1_* = *L_c_* ((*λ_1_* + 1)*A_1_* + *A_2_*), and *J_2_* = *L_c_* (*A_1_* + (*λ_2_* + 1)*A_2_*) (1)

*J_1_* and *J_2_* designate fluxes through affinities *A_1_* and *A_2_*, respectively, and *L_c_* represents the coupling conductance. Under totally coupled conditions (*λ_1_* = *λ_2_* = 0) both fluxes are equal. The dissipation function, *Ф*, of a coupled process is composed of two parts, *Ф_1_* for the output, and *Ф_2_* for the input reaction, with:

*Ф* = *Ф_1_* + *Ф_2_* (2a)

*Ф_1_* = *J_1_A_1_*, and *Ф_2_* = *J_2_A_2_* (2b)

*Ф_1_* = *L_c_*(*A_1_* + *A_2_*)*A_1_*, and *Ф_2_* = *L_c_*(*A_1_* + *A_2_*)*A_2_* (total coupling). (2c)

because *A_1_* usually is negative, *Ф_1_* must also be negative. Expanding the right hand terms yields:


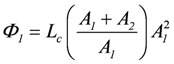
, and 
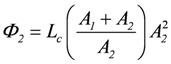
(2d)

The term: 


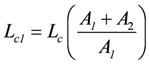
(2e)

represents that partial conductance of *L_c_*, which is associated with *A_1_*, while 


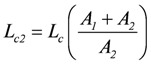
(2f)

belongs to *A_2_*. They relate to the usual different forms of energy being processed through the coupling reaction. Obviously, when *A_1_* is negative, *L_c1_* must also be negative to yield a negative *Ф_1_*. 

The same result can also be derived by starting from flux equations, yielding: 


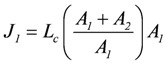
, and 
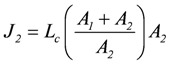
(3)

So, to yield a positive *J_1_*, *L_c1_* has to be negative for a negative *A_1_*. 

*A_1_* and *A_2_* are in series, hence, *L_c_* can be regarded as the equivalent conductance of both in series conductances *L_c1_* and *L_c2_*, yielding: 


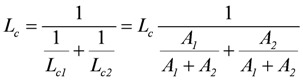
(4)

These theoretical results are confirmed by simulations. 

### 2.2. Conductances in Cycles between Coupled Reactions

In a reaction sequence in which two coupled reactions in series are involved, the output force of the first reaction, *A_1_^I^*, may be used by the second reaction as an input force *A_1_^II^*(*A_2_^I^* is the input affinity, *A_1_^II^* denotes the load affinity). In such a cycle between two coupled reactions, both forces must be equal but of opposite sign. The output power of the first reaction delivers the input power for the second reaction by flowing through *A_1_^I^*, and *A_2_^II^* = - *A_1_^I^*, and back to *A_1_^I^*(at zero power). At steady state, fluxes through *A_1_^I^* and *A_1_^II^* are equal, and hence, both dissipation functions of the cycle, *Ф_1_^I^* and *Ф_2_^II^*, must vanish. From *Ф_1_^I^* = - *Ф_2_^II^*, and *L_c1_^I^*(*A_1_^I^*)^2^ = -*L_c2_^II^*(*A_2_^II^*)^2^, *L^I^_c1_* = - *L^II^_c2_* is obtained. That is, the partial conductances of two coupled reactions in series are opposite and equal, if under conditions of steady state cycling the magnitudes of the output force of reaction *I* and the input force of reaction *II* are equal. 

*Ф_1_^I^* = -*Ф_2_^II^* can also be expressed in terms of the steady state flux of cycling and of resistances *R_c1_^I^* = 1/*L_c1_^I^*, and *R_c2_^II^* = 1/*L_c2_^II^*, yielding *R_c1_^I^J^2^* + *R_c2_^II^J^2^* = 0. It follows that at steady state cycling between two coupled reactions, the sum of the resistances in that cycle must vanish, *i.e.*, that the steady state flux through a cycle between two coupled reactions always occurs at zero overall resistance. Cycling is driven solely by *A_2_^I^* + *A_1_^II^*(*A_1_^II^* negative). Because both reactions are coupled, the conductance (resistance) of the whole cycling process is brought about by both partial (in series) conductances associated with the input and load affinity, respectively.

In oxidative phosphorylation (OP), as described in detail in [1], a proton cycle is generated over the inner mitochondrial membrane. At steady state, coupled outward proton pumping by redox (NAD_red_ and FAD_red_) reactions of the respiratory chain (*J_NA_* and *J_FA_*) equals the back flow of a given fraction (Q_H_) of protons through ATP synthase (*J_SY_*) and ATP/ADP exchange (*J_AE_*) plus H/Pi symport (*J_Pi_*). Both partial conductances, 


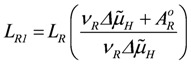
, and 
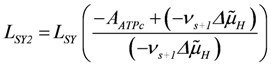
(5)

are opposite and equal. For the first reaction (index R) 
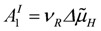
, and for the second 
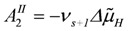
, with *v_R_* = *v_s+1_* (total coupling; *A_R_^o^* = affinity of *J_NA_* plus *J_FA_*; *v_R_* = *v_s+1_* = 4protons/extent of reaction).

The remaining fraction of protons (Qr_H_) flows back (driven by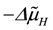) through several parallel conductances given by the proton leak flux, *J_PL_*, mitochondrial Na^+^/Ca^2+^ exchange (with Na^+^/H^+^ contracted to H^+^/Ca^2+^ exchange), 2*J_HCE_*, and the malate-aspartate shuttle, *J_MS_*. The partial conductance of this residual proton efflux and the sum of conductances of back flowing fluxes, are also of opposite equality.

Analogously, partial conductances of ATP cycling through the potentials of mitochondrial ATP (ATP_m_) production plus ATP transport (contracted to *A_1_^I^*), and of cytosolic ATP splitting (*A_2_^II^*) can be formulated. Opposite equality of partial conductances is also fulfilled for this cycle (the above results were obtained by using the simulation SIM_GlOx_ from reference [1]).

For a further illustration, an analytically solvable example is given in the Appendix section. Simple electric circuits consisting of one battery connected to an outer conductance, or of two batteries in series, are analysed. These examples show very clearly the behavior of coupled in series reactions. 

Further evidence of such an equality of conductances comes from the known fact that for a coupled reaction with an attached load, conductance matching (*L_Ld_* = *L_c_*) is needed to achieve a maximal power output [1]. At total coupling, the output power is given by:


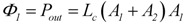
(6)

The maximal *P_out_* is found by differentiation with respect to the variable *A*_1_, while *A*_2_ remains constant, and by setting the derivative equal to zero:


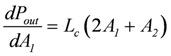
(6a)


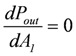
 leads to 
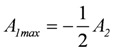
(6b)

Inserting *A_1max_* into equation (2e) yields *L_c1_* = - *L_c_* and because - *L_c1_* = *L_Ld_*, it follows *L_Ld_* = *L_c_*.

In addition to cycling at the inner mitochondrial membrane, other types of cycles occur in metabolism. Especially in skeletal muscle cells, the phosphofructokinase (PFK) reaction in conjunction with the fructose-1,6-biphosphatase (FBPase) operating anti-parallel represent a substrate cycle, which may control the pathway of glycolysis (GLY) more sensitively than would be possible by PFK alone. In this cycle, fructose-1,6-biphosphate (FBP), which is produced by ATP-coupled formation from fructose-6-phosphate (F6P), is cycled back via FBPase to F6P. However, usually both fluxes are not equal. Also to demonstrate the opposite equality of partial conductances for this kind of cycle, only equal fluxes can be used for this purpose. 

As a further example, the phosphocreatine shuttle will be considered. The creatine kinase (CK) reaction can also be regarded as a coupled reaction. Here, ATP splitting powers phosphocreatine (PCr) formation from creatine (Cr), which may proceed near equilibrium. As described in detail in reference [1], ATP is shuttled between locations of ATP formation (for instance in the inter-membrane space in mitochondria) and locations of high ATP demand like myofibrils. By analogy to an electric circuit built by two in series batteries with an outer circuit conductance (see Appendix (A4)), the output affinity of PCr formation in the inter-membrane space of mitochondria corresponds to *A_1_^I^* with associated *L^I^_c1_*, whereas the affinity of the reverse reaction in myofibrils corresponds to *A_1_^II^*(with *L^II^_c2_*). To ensure diffusional flow of PCr and Cr between both locations, an additional driving force (corresponding to U_e_; see (A4)) with associated conductance must be present. Under such conditions partial conductances do not match. Only when the additional conductance corresponding to the diffusional process (*L_e_*) is added to *L^I^_c1_* does this sum become opposite and equal to *L^II^_c2_*, as is shown in (A4). *L_e_* depends greatly on structural features. So, to achieve a high diffusional conductance, diffusional paths must be as short as possible, which in turn requires a high grade of structural organization [26,27,28]. 

It seems worth mentioning that coupled systems like pump and leak cycles are often not in a steady state. For instance, steady state cycling through sarco/endoplasmatic reticulum Ca^2+^ ATPases (SERCA) and Ca^2+^ release channels of the sarcoplasmatic reticulum (SR) breaks off during activation of contraction. There is an enormous Ca^2+^ efflux through release channels; meanwhile the pumping rate of SERCAs may be low. Under these conditions, respective conductances may greatly differ; however, when a new steady state cycling is reached, the partial conductance of SERCA must be opposite and equal to the conductance of the Ca^2+^ release channels. The opposite has to be expected, when release channels close again, and the Ca^2+^ pumping rate exceeds the release rate.

### 2.3. From Chemical Potentials to Mechanical Force Generation

In striated muscle cells like ventricular muscle cells (VMs) or skeletal muscle fibers (SMFs), force generation as well as shortening is brought about by the cyclic action of cross bridges. It is a known fact that this process is powered by ATP splitting. The underlying mechanism of the energy transduction process, however, is not completely understood. Here, a thermodynamic description of the cycle is derived using a formalism recently published [1]. It takes into account the basic energetics of enzyme-catalysed reactions, which states that the overall affinity of the catalysed and non-catalysed processes must be equal. For an enzyme-catalysed reaction like: 


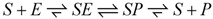


(S = substrate, E = enzyme, ES = enzyme-substrate complex, P = product), this means that at steady state the sum of the affinities of substrate binding, transition, and product release must yield the affinity of the non-catalysed reaction, which is given by the reaction affinity of all involved compounds in the bulk solution: 


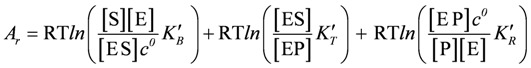
(7a)

or, after contraction of the first two terms:


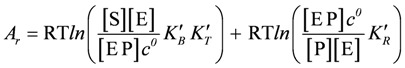
(7b)

yielding,


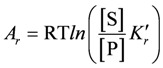
(7c)

With *K'_r_* = *K'_B_* × *K'_T_* × *K'_R_*(*k'_B_*, *k'_T_*, and ***k'_R_*** are equilibrium constants of the binding, transition, and release reaction, respectively, whereas *k'_r_* denotes that of the non-catalysed reaction). 

An analogous reaction sequence is used here to describe the cross-bridge cycle. The following cycle is given in chemical notation, *i.e.*, the charges of involved species are taken into account. The cycle begins with the splitting reaction of the de-energised actomyosin complex (A-M) by MgATP^2-^ in the diffusional space of myofibrils: 


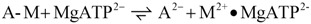
R_1_

This first reaction yields dissociated actomyosin with MgATP^2-^ bound to myosin (the bold point denotes binding to myosin). Two negative charges develop on the dissociated actin, which are neutralised by potassium ions, K^+^, stemming from free MgATP^2−^, which is now bound to myosin heads. On the dissociated myosin heads, it neutralises both emerging positive charges. This first actomyosin dissociation and binding of MgATP^2−^ to myosin is followed by ATP splitting on the myosin heads. This transition reaction is described by


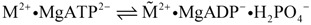
R2

It is coupled to the formation of energised myosin (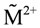), which is characterised by a tilting of the myosin head from a more bent arms position by an angle of about 60° towards the respective Z disc, so that now the myosin head builds a right angle with the opposing actin filament. 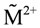 contains free energy from reaction R_2_ as conformational energy. 

The force generating stroke of the myosin head is triggered by the association reaction to form the energised actomyosin complex (cross-bridge):


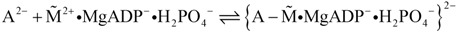
R_3_

Because of uncompensated charges, the resulting intermediate in curly brackets lacks firmness. A more stable conformation is obtained by the last reaction of the cycle, which restores the electro-neutrality of the cross-bridges: 


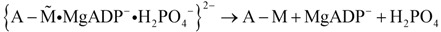
R_4_

The existence of a less stable interaction of myosin with actin has been shown previously [29,30,31]. 

In a coupled reaction, de-energised actomyosin is restored by dissociating first MgADP^-^ and H_2_PO_4_^-^ from cross-bridges and then by releasing the stored conformational energy. During this reaction the cross-bridge tilts back by 60° towards the sarcomere centre, whereby free energy is transferred to the actin filament as mechanical energy. 

From the above scheme (R_1_ to R_4_) two fluxes can be obtained, which are responsible on the one hand for the production of dissociated and energised myosin heads (*J_En_*), and on the other hand for the formation of cross-bridges and subsequent mechanical force generation by stroking (*J_Str_*). At steady state, a certain fraction of myosin heads of a half-sarcomere exists in a dissociated and energised state 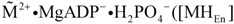, while the residual fraction interacts as cross-bridges with actin. The resulting fluxes are given by:

*J_En_* = *L_En_*(*A_En_^Ld^* + *A_En_^P^*), with (8a)

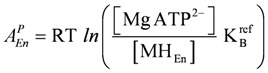
, and (8b)

*J_Str_* = *L_Str_*(*A_Str_^Ld^* + *A_Str_^P^*), with (8c)

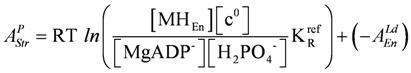
(8e)

(For a more complete description and definition of reference constants (K^ref^) see (A5); complete conductances (**L_En_** and **L_Str_**, respectively) are given in (A14) and (A15).)

If the constraint 
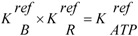
 is fulfilled, contraction of the affinities of both fluxes yields the overall affinity as required (*A_Str_^Ld^* + *A_ATP_*). Here **A_En_^Ld^** (stored as conformational energy) denotes the affinity coupled to binding of MgATP^2−^ to myosin heads (*A_En_^P^*), and **A_Str_^Ld^** the affinity which is coupled to the power stroke potential (*A_Str_^P^*). **A_Str_^Ld^** represents the mechanical work per mole of cross-bridges which has to be overcome during stroking. The quantity *J_Str_* × *A_Str_^Ld^* is directly related to mechanical power output *P_Str_* = *F_Ld_* × *v*(*F_Ld_* = load force in Newton (N) of all stroking cross-bridges of a given cross sectional area; *v* = velocity of shortening in m/s related to a given fiber length), which as such is conveyed to the surroundings. 

In the present model of the cross-bridge cycle, **A_ATP_** is used at two mechanistically and temporally separated steps. They are given on the one hand by binding of MgATP^2−^ and on the other hand by the release of MgADP^−^ and H_2_PO_4_^−^. Here, most of the free energy of ATP splitting is associated with *A_En_^P^*, which by the coupling process on myosin heads is transformed first into *A_En_^Ld^*, and then is delivered as *A_Str_^P^* to the power stroke after cross-bridge formation. Therefore, the stroke potential in mechanical units (cross-bridge force × stroke length × *N_A_*; *N_A_* = Avogadro’s number) must be equal to *A_Str_^P^* (see below). 

Because ionic species are involved, the reaction sequence of the cycle should be markedly enforced by electrostatic interactions. So MgATP^2−^ binding can proceed only if actomyosin dissociates, whereas release of products becomes possible only when at the same time cross-bridge formation occurs. Moreover, the conformational change in the myosin head forces it into a new position, which favours an interaction with actin at a new actin binding site displaced a certain distance towards the Z-disc. During stroking, binding of a new MgATP^2−^ molecule and detaching of cross-bridges may preferentially occur at the end of the power stroke, when cross-bridges form an angle of about 60° with the actin filament (see below for uncoupling by stroke shortening). 

The contractile performance of whole muscle and of SMFs is exceptionally well reproduced by Hill’s equation [19]. This equation relates the shortening velocity *v* to the mechanical load force *F_Ld_* which has to be overcome during shortening. 


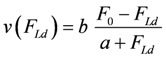
(9a)

The above function represents a hyperbola, which fits remarkably well with experimental data obtained under isotonic conditions. 

To obtain an equivalent expression from the flux equation **J_Str_**, the flux given in mM/s has to be converted into velocity with units of m/s. This is achieved by calculating the stroke frequency for a given concentration of stroking cross-bridges ([CB] = [CB]_tot_ – [MH_En_], in mM) and by multiplying with the stroke length *l_Str_*(in m) and the number of in series half-sarcomeres *N_hs_*. The result is:


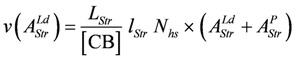
(9b)

**Figure 1:**
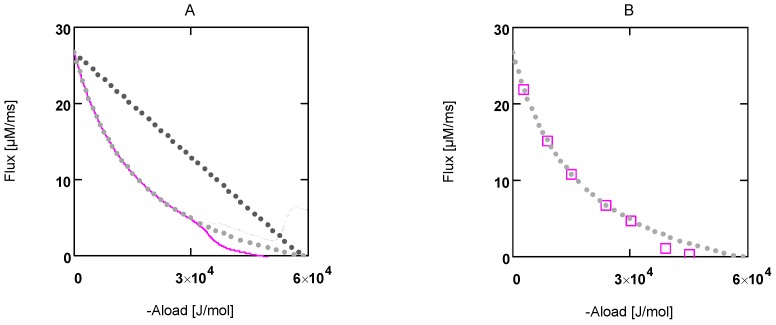
Flux as a function of load potential at 10.8 µM [Ca^2+^]. **A: **(grey dots) according to equation 9b; (light grey dots) according to equation 9c or 9d; (red line) according to equation 11a; (green line) according to equation 11b. **B:** (light grey dots) according to equation 9c or 9d; (red squares) results from simulation SIM*_GLYgen_*.

The above expression describes the shortening velocity as a function of *A_Str_^Ld^* at constant *A_Str_^P^*. It represents a straight line (Figure 1A). Introducing a Michaelis-Menten like inhibition factor associated with *L_Str_* yields the desired hyperbolic dependency: 


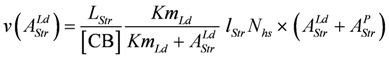
, or(9c)


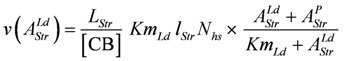
(9d)

Comparing equations 9a and 9d shows that the constant *b* of Hill’s equation is given by: 


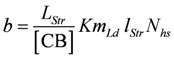
(in m/s) (9e)

As required, the quotient by which *b* is multiplied is dimensionless. To yield the shortening velocity as a function of force, *v*(*F_Ld_*), affinities and *Km_Ld_*(both in J/mol) have to be converted into units of force. This is achieved by dividing by *l_ Str_* and by multiplying by the molar number of cross-bridges. 

*A_Str_^Ld^* being negative, **F_Ld_** must also be ≤0. Expressing shortening velocity as a function of a positive variable yields with *F_Ld_* = - *F_Ld_^+^*


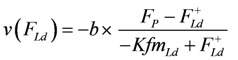
(10a)

**Figure 2:**
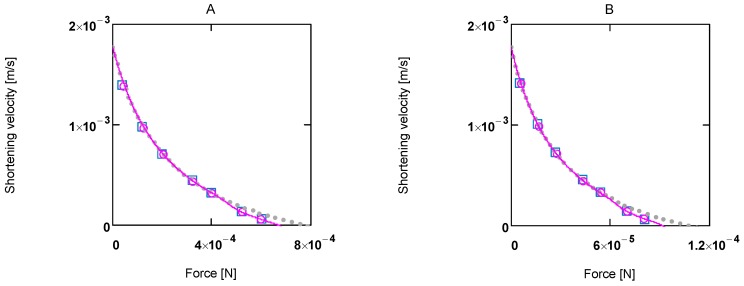
Shortening velocity as a function of load force at two different Ca^2+^ concentrations **A:**[Ca^2+^] = 1.08 µM; (light grey dots) according to equation 10b; (red line) equation 10b plus uncoupling; (red circles) results from SIM*_GLYgen_* versus load force; (blue squares) results from SIM*_GLYgen_* versus load force as sensed by cross-bridges; **B:** as in **A**, but at [Ca^2+^] = 0.36µM.

Setting - *Kfm_Ld_* = *a*, and - *b* = *b*^+^, gives (Figure 2.)


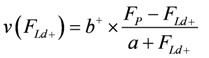
(10b)

The latter equation formally represents Hill’s equation. In that equation *F_0_* denotes the maximal force obtained under isometric conditions, whereas *F_P_* in the latter equation is obtained from the input affinity (*A_Str_^P^*) of **J_Str_** by converting it into units of force (see below for a derivation of *F_p_* ≡ *F_0_*). 

 When efficiency is represented as a function of shortening velocity, the experimental data follow a curved line with a maximal efficiency at 0.18 × *v*_max_ [12]. From NET it is known that such a maximum is produced by uncoupling. To create such a maximal efficiency, uncoupling terms have to be incorporated into **J_Str_**. Variable, load dependent *λ* values (*λ*(*A_Str_^Ld^*) instead of constant *λ's*) are defined, to preserve the hyperbolic nature of the function. In this way, uncoupling becomes operative only when *A_Str_^Ld^* exceeds a certain value (Figure 1A). Both flux equations are given by:


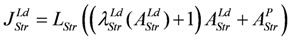
(11a)


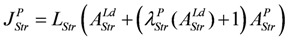
(11b)

These latter equations (for a complete description, especially of conductances, see (A15)) appear in simulations. *λ* values are given as functions of *A_Str_^Ld^*, e.g.,


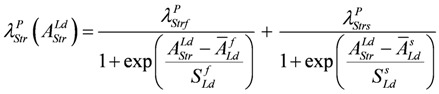
(12)

(see below for a mechanistic interpretation of uncoupling and *λ* values). Conversion to mechanical units can then be done in the same way as shown above. 

In Figure 1 the effects of uncoupling are shown. At a load of about −3.0×10^4 ^ J/mol, deviations from the hyperbolic (coupled) curve begin to arise. From the plots it can be seen that uncoupling leads to a shift of the intersection with the abscissa to less negative values of *A_Str_^Ld^*, whereas - (*J_Str_^P^*) is still maintained, even at *A_Str_^Ld^* = - (*A_Str_^P^*), where the coupled flux must be zero and only uncoupled fluxes are possible. 

In the following, an attempt has been made to interpret the above results, which were gained from a phenomenological approach, mechanistically by relating coupled and uncoupled fluxes to possible cross-bridge actions. 

At *A_Str_^Ld^* = - (*A_Str_^P^*), coupled reactions with associated actin filament movement come to a halt, because the driving force has vanished. As already mentioned above, now only uncoupled fluxes can occur. Such a situation may also be realised with isometric contraction, which is known to be associated with ATP splitting and heat production, but without power output. That is, a mechanism has to be found which explains the identity of the isometric force *F_0_* with *F_P_*, which was merely formally derived from the input affinity *A_Str_^P^* by a conversion factor. This is achieved by defining the uncoupling mechanism by a shortening of the stroke length *l_Str_* of the power stroke. Total uncoupling is reached when **l_Str_** = 0. This may be realised under isometric conditions. Free energy corresponding to *A_Str_^P^* ≈ *A_ATP_* is delivered to actin filaments as mechanical work, i. e., *F_0_* × *l_Str_* × *N_A_* = *A_Str_^P^*. Shortening may be brought about through splitting of actomyosin bonds before the whole stroke length is transferred to an actin filament. When *A_Str_^Ld^* = - *A_Str_^P^*, as is realised under isometric conditions, actomyosin splitting already occurs at zero stroke length, so that no energy can be delivered to the actin filaments. Only force development by cross-bridges during the time interval between bond formation and bond splitting is possible under these conditions. This may be achieved by the torque every myosin head exerts on an actin filament after bond formation and release of H_2_PO_4_^−^ and MgADP^−^. The associated force then acts on these filaments, but without being able to bring about filament movement, since this is hindered by the equal and opposite load force. Therefore, if the force remains constant over the whole stroke length, then *F_P_* is equal to the isometric force *F_0_*.

When compared with a cross-bridge cycle during contractions, the cross-bridge cycle under isometric conditions becomes altered insofar as coupled stroking is impossible; the power stroke occurs completely uncoupled, so that all free energy associated with *A_Str_^P^* becomes dissipated as heat. Moreover, dissipative stroking under these conditions may occur in the presence of bound MgATP^2−^. The following derivation shows how stroke shortening may be involved with uncoupling.

Stroke shortening is given by: 


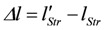
(negative)(13a)

this leads to,


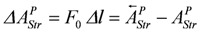
(at constant force)(13b)

Under totally coupled conditions, the input flux is given by: 


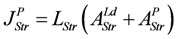
(13c)

Uncoupling by stroke shortening dissipates free energy, which can be expressed by a leak dissipation function:


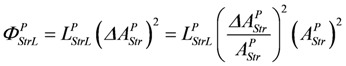
(13d)

The leak conductance *L^P^_StrL_* can be replaced by **L_Str_**, because this latter conductance may depend mainly on the formation mechanism of the actomyosin bond. The stroke reaction associated with conformational changes of the myosin head is assumed to proceed at a high conductance, since the energising reaction (**J_En_**), which is coupled to the same conformational change in the reverse direction, also proceeds at a very high conductance. So an increase by stroke shortening of a high conductance (stroking) in series with a low conductance (bond formation) may be negligible, so that *Ф^P^_StrL_* can be expressed as:


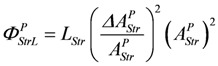
(13e)

Comparing this latter equation with that used in the simulation, 


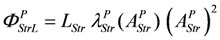
, yields:


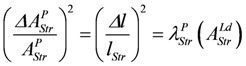
(13f)

The input flux then is given by:


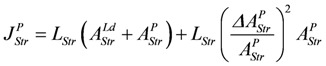
(13g)

The output flux is reduced by stroke shortening as if it were uncoupled. The same dissipation function *L_Str_*(*ΔA_StrL_^P^*)^2^ is associated with output reactions, yielding: 


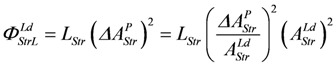
(13h)

and the output flux: 


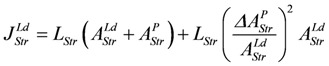
(13i)

It follows,


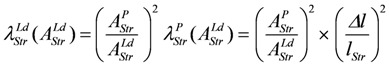
(13j)

For *Δl* = 0, identical coupled fluxes arise, and for *A_Str_^Ld^* = - *A_Str_^P^*, both *λ* values are equal. Moreover, if *λ_Str_^Ld^* = *λ_Str_^P^* = 1, (*Δl/l_Str_*)^2^ is also equal to 1.0, which means that now isometric conditions do exist. 

From equation (13i) it can be taken that uncoupling by stroke shortening reduces *J_Str_^Ld^* as if there were a leak flux through *A_Str_^Ld^*. On the other hand, *J_Str_^P^* increases (equation (13g)) as if there were an additional leak flux through *A_Str_^P^*. The above derivations demonstrate that stroke shortening obviously leads to the same effects as uncoupling by leak fluxes. It seems justified, therefore, to also describe uncoupling by stroke shortening by lambda values, as was done previously mainly in the context of oxidative phosphorylation.

The degree of coupling is given by,


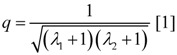
(14a)

with above results this yields:


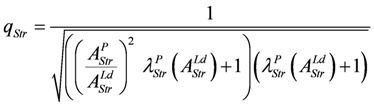
(14b)

Under the limiting conditions of isometric contraction (*A_Str_^Ld^* = - *A_Str_^P^*; (*Δl/l_Str_*)^2^ = 1.0), *q_Str_* is given by:


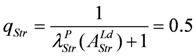
(14c)

At loads ≈ -3 × 10^4^ < **A_Str_^Ld^** < −*A_Str_^P^*, *λ_Str_^P^*(*A_Str_^Ld^*) will be smaller than 1.0 but not zero, so that the process in this region would proceed at a higher degree of coupling (e. g. at *A_Str_^Ld^* = −4.5 10^4^, *q_Str_* = 0.833). At values of *A_Str_^Ld^* > ≈ -3 × 10^4^ the process is totally coupled ((*Δl/l_Str_*)^2^ = 0), that is, cross-bridges work at full stroke length. Only this part of the performance curve (Figure 1 and Figure 2) is hyperbolic and fulfils Hill’s formalism. Between the intersection (**A_Str_^Ld^** = −4.756×10^4^) and *A_Str_^Ld^* = - *A_Str_^P^*, *J_Str_^Ld^* formally could be negative, which would mean that actin filaments were moving in the direction of stretching. This is, however, impossible, because actomyosin bonds would have to be broken by a load force, which is smaller than *F_0_*. Therefore, in this region of loads, *J_Str_^Ld^* cannot be negative; it must remain zero. 

### 2.4. Power Output and Efficiency

**Figure 3:**
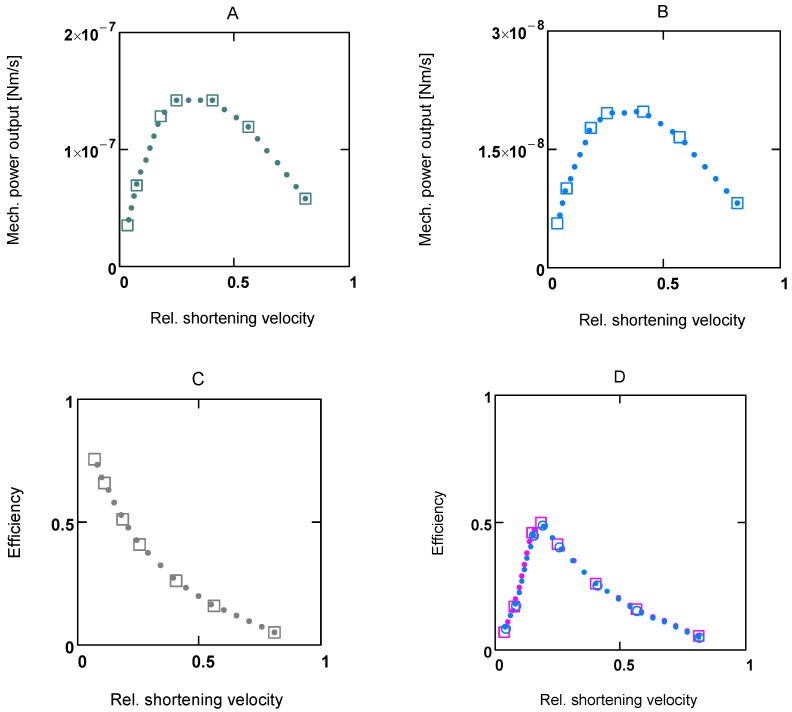
Power output and efficiency at two different Ca^2+^ concentrations. (**A)** and **(C)** [Ca^2+^] = 1.06 µM; **(B)** [Ca^2+^] = 0.36 µM; **C:** under totally coupled conditions; (**D)** (red squares) efficiency at 1.06 µM [Ca^2+^]_, _(blue circles) efficiency at 0.36 µM [Ca^2+^]. All plots are results from SIM*_GLYge_*.

In experiments, mechanical power output is often represented in relation to shortening velocity. In Figure 3, power and efficiency plots at two different [Ca^2+^]s (1.08 and 0.34 µM, respectively) are shown. Respective curves have similar shapes; however, *F_0_* and *v_max_*, and therefore power output values, are markedly increased at high [Ca^2+^]. 

Efficiency curves at both [Ca^2+^]s are nearly identical (Figure 3D). In panel B, efficiency of a totally coupled cross-bridge cycle is shown. Under these conditions the curve has no maximum. 

Partial conductances can be calculated from **L_En_**, *A_En_^Ld^*, and *A_En_^P^*, as well as from *L_Str_*, *A_Str_^Ld^*, and *A_Str_^P^*. All results derived in the above sections could be verified by the simulation (SIM*_GLYgen_*). So,


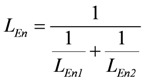
, and 
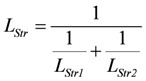
. (15)

also, *L_En1_* = -*L_Str2_* is fulfilled, and therefore, cross-bridge cycling at zero resistance.

In addition, the equality of 


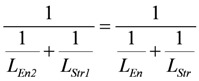
(16)

which describes the conductance of the whole cycle including coupled inputs and outputs is nearly exactly obeyed.

The overall efficiency of the cross-bridge cycle is obeyed: 


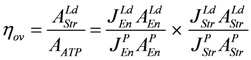
(17)

as is the overall dissipation function given by:


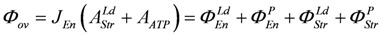
(18)

Figure 3D shows efficiency curves at 1.08 and 0.36 µM [Ca^2+^]_._ They are very similar; their maximum lies at about 0.18 *v_max_*. Because the appearance of the maximum is caused by uncoupling, the coordinates of *η_max_* are highly dependent on uncoupling parameters. 

### 2.5. Calcium Ions and Force Development

In the previous section it was shown, how shortening velocity depends on **A_Str_^Ld^** at a given [Ca^2+^]. On the one hand, the driving force is changed by the load potential (see Figure 1, linear dependence), and on the other hand the conductance *L_Str_* depends on **A_Str_^Ld^** through the hyperbolic inhibition factor. At a given [Ca^2+^]_, _ both effects are responsible for the characteristic appearance of the performance curve under coupled conditions. 

**Figure 4:**
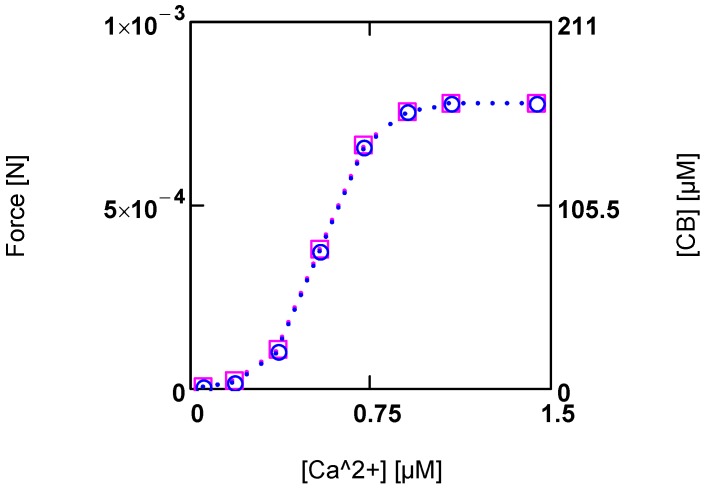
Developed force and cross-bridge concentration [CB] and their dependence on [Ca^2+^]. (red squares) force; (blue circles) [CB]. Notice that at the given dimensioning of the right ordinate a matching of results is obtained.

In the present model of the cross-bridge cycle, interference of [Ca^2+^] with *A_En_^P^* as well as with *L_Str_* is necessary. In the latter case, [Ca^2+^] can activate *J_Str_* through a sigmoid activation factor (A15). This takes into account the fact that Ca^2+^ binding to troponin C removes the inhibition of cross-bridge cycling, so that binding of myosin heads to actin binding sites becomes possible [32,33]. On the other hand, [Ca^2+^] is known to strongly activate force development. Here it is assumed that this may be caused by an increase in cross-bridge concentration [CB]. By introducing a [Ca^2+^] dependent *K_B_^ref^* (see (A14)), a sigmoid variation in both [CB] and force *F* by [Ca^2+^] can be obtained (Figure 4.). 

At steady state, a certain [CB] is produced by [Ca^2+^] activated *J_Str_*, and in addition by [Ca^2+^] inhibited *J_En_* (see (A14)). The inhibition of *J_En_* by [Ca^2+^] is brought about by a decrease of *A_En_^P^* with increasing [Ca^2+^]. This is possible because this reaction proceeds at a very high conductance and therefore, is close to equilibrium. So already a small variation of the driving force can produce a large change in the reaction velocity. In this way, a sensible, [Ca^2+^] dependent adjustment of [CB] and force can be achieved. An elevation of [Ca^2+^] thus increases both shortening velocity as well as force development. 

The total myosin head concentration ([MH_En_] + [CB]) of a half-sarcomere amounts to 656 µM (see Methods). At a saturating [Ca^2+^] of 1.08 µM, fluxes *J_En_* and *J_Str_* are so adjusted as to yield a concentration of [CB] = 0.25 ([MH_En_] + [CB]), *i.e.*, at this [Ca^2+^], 25% of myosin heads form cross-bridges and thus are involved with cycling and force generation. At [Ca^2+^] = 0.36 µM, only about 3% of cross-bridges are engaged, and at 0.09µM [Ca^2+^], [CB] is further markedly reduced, which means that now near resting conditions are reached.

It seems plausible to suggest that during shortening it is not always the same group of cross-bridges that is active, but that, e.g., at 1.08 µM [Ca^2+^], four different groups may alternately be involved with contraction. The cycling frequency of an individual cross-bridge would then be much lower than the frequency of ATP splitting, which might be advantageous, especially at high velocities. Furthermore, an alternating involvement of groups may be absolutely necessary for a smooth shortening. How this might be accomplished is so far not known. An involvement of special filaments of the sarcomere cytoskeleton [34,35,36], which may be responsible for a subtle sensing of load forces and an undisturbed takeover of a given load by a new fraction of cross-bridges during synchronous stroking, seems indispensable. 

The values of maximal tension (=force/unit area in N/m^2^ = Pa, Pa = Pasqual) obtained from SIM*_GLYgen_* (A16) in the present study are comparable to experimental values. For instance, a value of 372 kPa (from *F_0_* = 7.756 × 10^−4^ N, [Ca^2+^] = 1.06 µM, 37°C) found here, seems to be in reasonable agreement with about 320 kPa resulting from measurements with a fast-twitch mouse fiber at 25°C [37]. The extrapolated value of maximal shortening velocity, *v_max_*^HS^ = 1.95 µm × HS^−1^ × s^−1^ ([Ca^2+^]= 1.06 µM, *A_L_* = 0 J/mol, HS = half-sarcomere) compares to 1.6 µm × HS^−1^ × s^−1^ of frog fibers at 0°C [12]. A value of *η_max_* of about 50% at about 0.18 *v_max_* ([Ca^2+^] = 1.06µM) results from adjustment. It compares to the experimental values of 35–45 % for the same value of *v* for frog muscles at 0 °C [12]. 

All these parameters of contractile performance may, however, be reduced to a certain extent by dissipative frictional processes associated with *v* which are not addressed in the present simulation. Such dissipation during fiber shortening may be produced mainly by viscous deformations of membranes and the filament lattice.

### 2.6. [H^+^], [Mg^2+^], and Fatigue

Enzyme-catalysed ATP splitting by myosin heads is formulated here with respect to the ATP species MgATP^2−^. By using a reference constant and binding polynomials, an [H^+^] and [Mg^2+^] dependent *K'_ATP_* of this reaction can be formulated (see A6 and A7). In simulations of fatigue, in addition to [H^+^], [Mg^2+^] has also been included as a variable, especially because this ion may interfere with ATP species and so may influence *J_En_* through a change in [MgATP^2−^], which in turn would alter [CB]. 

Changes in [H^+^] in the sarcosol are brought about mainly by two different mechanisms, which are both related to metabolic activity. One source of protons is manifest when metabolism is switched from rest to high power output. Fluxes in ATP consumption and production, *J_ATP_^Con^* and *J_ATP_^Pro^*, respectively, must then both increase to the same extent to reach a new steady state. During the adjustment, a phase of disturbed steady state occurs, during which both fluxes do not match. When power output increases, *J_ATP_^Con^* always leads *J_ATP_^Pro^*, *i.e.*, there is an uncompensated ATP splitting until a new steady state is reached, at which point ATP production again equals ATP consumption. 

According to Alberty [20], this reaction is associated with proton production in dependence of [H^+^] and [Mg^2+^] (see (A6) and (A7)) for derivation of [H^+^] changes and pH buffering). In addition, the CK and adenylate kinase (AK) reactions are involved, because these equilibria are also changed under these conditions and, as with ATP splitting, H^+^ and Mg^2+^ binding species are involved. Buffering of both ion concentrations is brought about mainly by sites intrinsic to the sarcosol. For Mg^2+^ binding sites, an additional release of Mg^2+^ by interfering [H^+^] has to be expected. 

**Figure 5:**
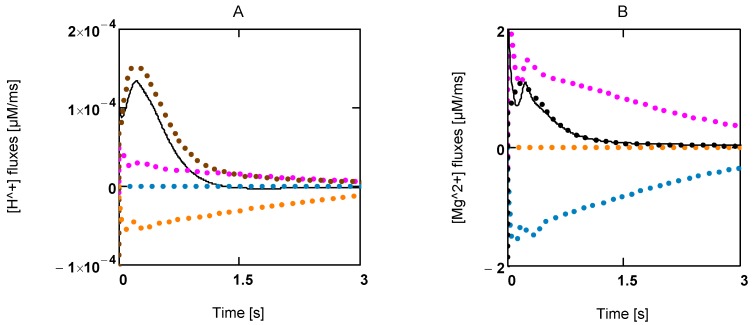
Time courses of [H^+^] and [Mg^2+^] during extreme power output. **A:** [H^+^] fluxes; (brown) mainly LDH reaction and lactate transport; (red) ATP splitting; (blue) *J_AK_*; (yellow) *J_CK_*; (black line) resultant [H^+^] flux; **B:** [Mg^2+^] fluxes of the same reactions.

Figure 5A shows the time courses of rates of [H^+^] changes. Interestingly, [H^+^] production by ATP splitting is practically compensated by [H^+^] consumption by the CK reaction. The contribution by the AK reaction is negligible. A similar behavior is found for Mg^2+^ (Figure 5B). A concentration increase in this ion is mainly brought about by acidification.

A second source of protons is given by the disturbance of lactate production by glycogenolysis (or glycolysis) and lactate efflux via lactate/H symport at the sarcolemma. Especially when lactate and H^+^ accumulate in the glycocalyx (the outer aspect of the sarcolemma), the concentrations of these compounds also increase drastically in the sarcosol. This seems to be the main mechanism of sarcosolic acidification. 

Muscular fatigue at the cellular level can be defined as a phase of markedly reduced contractile performance, which largely recovers after a period of rest [38]. Because metabolites like creatine, ADP, Pi, H^+^, and lactate accumulate during conditions of fatigue in a similar way as can be observed during ischemia or hypoxia, which are known to be the result of impaired ATP production, it seems justified to suggest that the preconditioning for fatigue may also be initiated by a deterioration of the energy metabolism of the muscle fibers. Whenever ATP delivery does not match ATP consumption, such a situation may arise. 

These effects can be easily demonstrated with a simulation of glycogenolytic or glycolytic ATP production in the absence of mitochondrial metabolism (SIM*_GLYgen_*_,_ see (A16)), which is related to the energy metabolism of fast muscle fibers. At 1.08 µM [Ca^2+^] and a load of –1.5 × 10^4^ J (constant glycogen content and glucose concentration [Glu] = 4.0 mM), efficiency of glycogenolytic ATP production is *η_GLYgen_* = 0.722, that of glycolytic ATP *η_GLY_* = 0.525. The higher efficiency is mainly caused by the stoichiometric coefficients of coupled ATP production of 3.0 and 2.0 for the glycogenolytic and glycolytic pathways, respectively. 

Under these conditions of high power output, metabolite concentrations change only moderately compared to resting conditions (at 1.06 µM [Ca^2+^] and a load potential of −1.5 × 10^4^ J/mol, [ADP] = 113, [Pi] = 8.32 × 10^3^, phosphocreatine concentration [PCr] = 9.7 × 10^3^, lactate concentration [Lac] = 3.0 × 10^3^, [Mg^2+^] = 832, and pH = 7.09). 

However, when a back pressure on glycogenolysis (or glycolysis) is produced by accumulated extracellular [Lac]_e_ and [H^+^]_e_, the flux through this pathway may become reduced. In addition, efficiency has been reduced by switching from glycogenolysis to glycolysis. The power output of ATP production is markedly reduced by these combined effects. As a result, the power of ATP production begins to fall, so that ATP consumption may overcome ATP production. Steady state cycling through ATP consuming and producing pathways can now no longer be maintained. 

**Figure 6:**
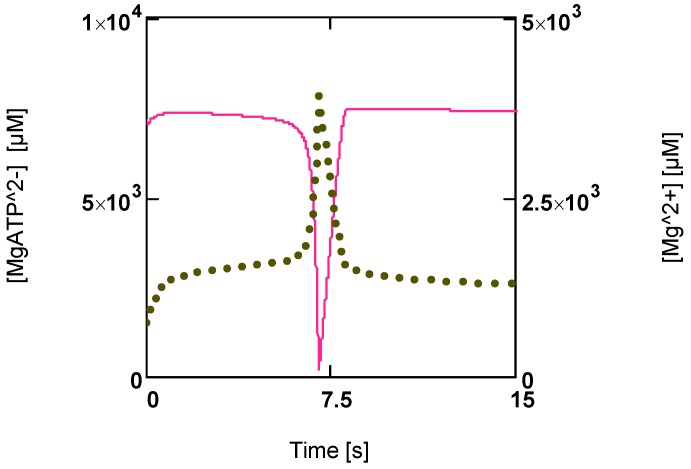
Time courses of [MgATP^2−^] and [Mg^2+^] during development of fatigue. (red line) [MgATP^2−^]; (green points) [Mg^2+^].

Figure 6 shows that a first phase of slowly falling [MgATP^2−^] is followed by a phase of continuously enhanced reduction of this ATP species to low values ([MgATP^2−^] = 230.0 µM; [PCr] = 1.6 µM). Immediately after reaching a minimum, a rapid recovery of [ATP] (up to starting values) begins. [Mg^2+^] shows a corresponding behavior. During the first phase it increases because of acidification, and then a sharp peak is produced by the onset of an extreme uncompensated ATP splitting (Figure 6). An increased [Mg^2+^] may counteract the switch off of cross-bridge cycling and may aid recovery by increasing [MgATP^2−^].

Other parameters such as [PCr], [Pi], [Lac], and pH only partially recover under these conditions of extreme power output. An almost complete recovery, however, is possible under conditions of markedly reduced power output near resting [Ca^2+^]. 

**Figure 7:**
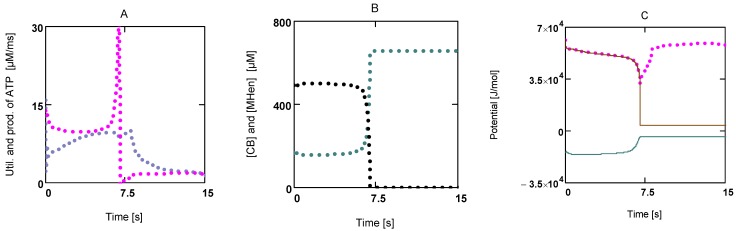
Time courses of *J_ATP_^Con^* and *J_ATP_^Pro^*, of [CB] and [MH_En_], and potentials of the cross-bridge cycle during fatigue development. (**A)** (red) *J_ATP_^Con^*; (blue) *J_ATP_^Pro^*; (**B)** (black) [CB]; (blue) [MH_En_]; (**C)** (red points) *A_ATP_*; (brown line) *A_Str_^P^*; (blue line) *A_Str_^Ld^*; notice that after 7.5 s *A_Str_^P^* and *A_Str_^Ld^* become opposite and equal.

How this switch back to normal [ATP] is brought about can be seen from Figure 7. Not only have fluxes of ATP consumption and production, *J_ATP_^Con^* and *J_ATP_^Pro^*, become different now (*J_ATP_^con^* > *J_ATP_^pro^*; Figure 7A), both fluxes of the cross-bridge cycle, *J_En_* and *J_Str_*, have also changed. These fluxes determine concentrations of [MH_En_] and [CB], respectively. An increase in *J_En_* and a decrease in *J_Str_* would lower [MH_En_] (whereby [CB] would be increased). Both concentrations always change reciprocally (Figure 7B). *A_Str_^P^* and *A_Str_^Ld^* are also affected. *A_Str_^P^* in particular is rapidly reduced until it is equal to −*A_Str_^Ld^*. Now all fluxes of the cycle must vanish, because the driving force of *J_Str_* has become zero. As a result, ATP consumption by cross-bridge cycling is switched off.

Under these conditions all myosin heads form cross-bridges, which however are unable to perform the power stroke, since the input force is equal to the opposed load force. In such a situation myosin heads may be bound to actin and may have dissociated H_2_PO_4_^−^ and MgADP^−^ similar to an isometric contraction, but in contrast to those latter conditions, equilibrium of forces is now brought about at a much lower load force (*A_Str_^P^* = ^−^*A_Str_^Ld^* = 0.375 × 10^4^ J/mol at 1.08 µM [Ca^2+^]). A load-dependent actomyosin splitting by MgATP^2−^ at the beginning of the stroke, that is uncoupling, is impossible under these conditions. So cross-bridge cycling with concomitant ATP consumption may be completely prevented. [ATP], therefore, can recover rapidly, even if the conditions leading to fatigue first remain unchanged.

By this mechanism the fatigued skeletal muscle fiber is capable of protecting itself from the dangerous risk of irreversible cell damage. This seems to be necessary, since this cell type is voluntarily controlled without any protecting mechanism against an unbridled consumption of ATP, as is known to occur with other ATP coupled reactions such as, for instance, ion pumps. These are controlled mainly by the ion concentration that they are transporting. For example, when [Na^+^] in the sarcosol is lowered by the Na/K pump to values below 10.0 mM, the reaction rate of this transport process is increasingly deactivated by the decreasing [Na^+^], so that ATP consumption also is reduced.

Such a protective mechanism is not known, however, for the cross-bridge cycle. Contractions with concomitant ATP splitting would be incessantly initiated, as long as firing of nervous impulses persisted. The voluntary muscle fiber would obey this parent command up to exhaustion or even up to cell death, if the fatigue producing mechanism were absent. Obviously it represents that special control mechanism which is necessary to protect voluntary muscle from dangerous ATP depletion during phases of high energetic demands. 

The above results are obtained from a simulation, in which ATP production is confined solely to glycolysis. A whole muscle, however, is constructed from many types of functionally different fibers, with slow fibers having densely packed mitochondria, and fast fibers in which mitochondrial density can be very low. It is a known fact that especially fast fibers with a very low content of mitochondria and, therefore, a mainly anaerobic ATP production, are much more liable to be affected by overpowering than slow fibers. This may be brought about primarily by the preconditioning effects especially associated with this fiber type. At very high energetic demands, fast fibers produce much more lactate and protons through glycolysis than slow fibers, which can oxidise pyruvate by mitochondria. That is, the glycolytic ATP production rate of fast fibers may be decelerated by a back pressure, which may be generated by accumulating lactate and protons during high power output. In slow fibers with a high rate of oxidative glucose metabolism, such a back pressure cannot be produced as easily. Therefore, the metabolic changes leading to fatigue are simulated here with respect to fast fibers with a low resistance to fatigue. This weakness may be best demonstrated with an extreme fiber type, which can produce ATP solely by GLY. However, fibers completely devoid of mitochondria may not exist in vertebrate muscle. The results of this fatigue model, therefore, can only be taken as an approximation of real fast muscle fibers. Muscular contraction on the basis of NET has been treated theoretically by Caplan and Essig [13]. These authors explained the curvature of Hill’s equation by an uncoupling. They defined the degree of coupling by 
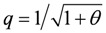
 with *θ* = *a*/*F_0_* = *b*/*v_max_*(*θ* between 0.2 and 0.3). This latter equality is also fulfilled by the present model (***θ*** = 0.309). However, in contrast to the approach of the above authors, the hyperbolic form of Hill’s equation is produced here by introducing a Michaelis-Menten-like inhibition factor into the respective conductance, as already mentioned above. Moreover, uncoupling in the present model is produced through a load-dependent stroke shortening, which generates the maximum obtained with power plots. 

## 3. Methods

Here the energy metabolism of a fast twitch muscle fiber is treated. That is, ATP production by this fiber type is solely brought about by metabolism of glycogen and/or glucose. Mitochondria are absent. Glycolytically produced [NAD_red_] has to be reoxidised by the lactate dehydrogenase (LDH) reaction, and the lactate plus proton formed thereby is released to the extracellular space via Lac/H symport. Electrophysiological reactions at the cell membrane (sarcolemma) are omitted. Also, most reactions of the sarcoplasmatic reticulum (SR) are not addressed. Only Ca^2+^ pumping by the sarco/endoplasmatic reticulum Ca^2+^ ATPase (SERCA) as an ATP consuming reaction is included in simulations besides several other reactions of ADP production (see SIM*_GLYgen_* (A16)) taken over from reference [1]. Therefore, [Ca^2+^] is treated as an adjustable constant. 

To determine the fractional fiber volume *V_Cell_*, a cylindrical geometry of the muscle cell is assumed. With radius *R_Cell_* = 25.76 µm, and a length *L* = 10^3^ µm (fraction of whole fiber length), *V_Cell_* = 2.0847 × 10^6^ µm^3^ or 2.0847 nL, and *A_Cell_* = 2.0847 × 10^3^ µm^2^. From data of Aliev *et al.* [39] for the heart, the volume of the sarcosol, *V_c_*, can be determined by adding the mitochondrial to the fibrillar volume, yielding *V_c_*/*V_Cell_* = (321 + 195 + 55)/758.5 = 0.7528 or *V_c_* ≈1.57 nL. Then *α_c_* can be obtained using *α_c_* = 10^−12^/(F×*V_c_*) = 6.6024×10^−9^ µM/C (F = Faraday’s constant, C = Coulomb). That is, to yield the corresponding flux in µM/ms from an electric current entering the sarcosol, this current in fA (= pS×mV = 10^−18^ C/ms; pS = pico Siemens) has to be multiplied by *α_c_*. 

For calculation of force and velocity, the dimensions of the force generating cross-sectional area and the number of half-sarcomeres (HS) of the fibrils must be known. The contractile machinery of skeletal muscle fibers is organised in fibrils having diameters between 1.0 and 2.0 µm, which are built up from in series sarcomeres connected by Z-discs over the whole length of a fibril, *i.e.*, from end to end of the fiber. The functional unit is given by the HS. The principal filaments of an HS are the thick myosin and the thin actin filaments. In cross-sections, myosin filaments show hexagonal geometry. From this symmetry the fibrillar volume *V_Fibr_* can be obtained. One hexagon is composed of six equilateral triangles of side length *l_Tri_* = 41.0 nm [12] and equal angles of 60°. The area of a hexagonal fibril (or HS) of radius *R_Fibr_* = 18.0 × 41.0 = 738 nm is given by: 


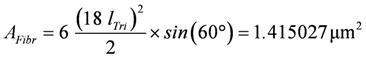
, and(19a)


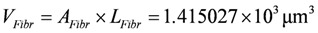
 (=1.415027×10−15 m3 or 1415.027 pL) (19b)

The total volume of fibrils is given by 0.866×*V_Cell_* (see reference [39]). The number *N_Fibr_* is then given by: 


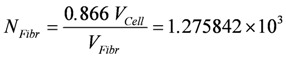
(19c)

For the determination of the total number of myosin heads of an HS, the number of myosin filaments of an HS must be known. The above hexagonal area of an HS can be constructed from equilateral triangles of *l_Tri_* = 41.0 nm. A large triangle of the hexagonal HS with n-fold side length contains: 


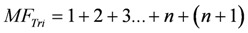
(20a)

corner points representing myosin filaments. All points of the HS hexagon are given by: 


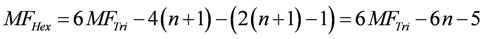
(20b)

For an 18-fold increase of *l_Tri_* from 41.0 nm to 738 nm (*n* = 18), the resulting HS hexagon contains, with *MF_Tri_* = 190, *MF_Hex_* = 1027 myosin filaments per fibril. 

Myosin filaments contain 294 myosin heads per half filament. In an HS, thus 294 × *MF_Hex_*, and in the whole fiber:


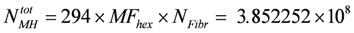
(20c)

myosin heads are contained. 

Their concentration is obtained by dividing by *N_A_* (= 6.022142 × 10^23^ particles per mol), and by the volume of the water diffusible space in the filament lattice containing myosin heads of all HSs of the cross-sectional area of a fiber, *V_Lat_*. This volume is given by: 


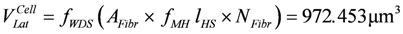
or 0.972453 pL(20d)

(*l_HS_* = HS length at rest = 1.1 µm, *f_MH_* = length fraction of myosin filament containing myosin heads in terms of HS ([12]) = 0.62364, *f_WDS_* = volume fraction of water-diffusible space in the filament lattice volume = 0.7852). 

The myosin head concentration is given by:


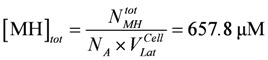
(20e)

At steady state, the concentration of stroking cross-bridges, [CB] = [MH]_tot_ – [MH] ([MH] = the remaining myosin head concentration given by the simulation) is determined by two fluxes (see Results). It is adjusted to about 25% of [MH]_tot_ at [Ca^2+^] = 1.08 µM and a load potential *A_L_* = 3.0×10^4^ J/mol. The force generated by [CB] is given by:


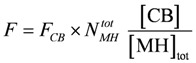
(21a)

*F_CB_* is the force of one single cross-bridge. It is obtained here from the stroke input potential *A_P_* (see Results) under these conditions, by assuming that the stroke length *l_Str_* = 12.0 nm. Then *F_CB_* is given by: 


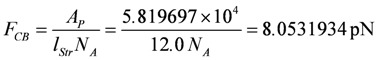
(21b)

per cross-bridge, yielding *F* = 0.7735 mN per fiber, and a tension of 3.71 × 10^5^ Pa (related to *A_Cell_*, Pa = Pasqual). The force is generated at 1.08 µM [Ca^2+^] by about 25% of myosin heads ([CB] = 164 µM). All force-generating cross-bridges are contained in the parallel HSs of the cross-sectional area of 1027 fibrils of one muscle fiber. 

Simulations were solved using Mathcad^®^ 14.0 or 15.0 M011 solver *AdamsBDF*. The programs were run under Microsoft^®^ Windows 7 and XP Professional. 

## 4. Conclusions

Cycling between coupled reactions occurs, especially in energy metabolism. It is shown that the overall resistance of such cycles must vanish, and that the resistance or conductance associated with the negative output affinity of a coupled reaction also has to be negative. The following may be illuminating: The entropy change of a spontaneously proceeding reaction (Δ*_r_S_i_*) is always positive. When a reaction is forced against spontaneity, Δ*_r_S_i_* must become negative. All reaction parameters associated with entropy changes, like affinities and conductances, must inevitably follow a sign change of Δ*_r_S_i_* whenever such a change occurs. This is not a contradiction to Ohm’s law, but a consequence of the phenomenological definition of a conductance through ±*L* = *J*/±*A*. It can be concluded that the occurrence of negative conductances is realised not only with biochemical reactions in living cells, but represents a fundamental concept of coupled processes.

This concept is realised here for the cross-bridge cycle. The reactions of the cycle are described on a thermodynamic basis using the kinetic approach of enzyme-catalysed reactions. Hill’s equation for muscular performance can be derived on this basis. However, uncoupling has to be introduced to yield a maximal efficiency of power output. Here the uncoupling mechanism is not an accidental process during energy transduction, but a necessary interference during force generation, which ultimately produces an isometric contraction. 

Although mechanical acceleration may also be possible on a cellular basis by changes in sarcosolic [Ca^2+^], it seems highly unlikely, however, that this may be sufficient to allow normal locomotion of a subject. Only the control by the nervous system can bring about coordinated actions of several muscle fibers, groups of fibers, or even several different muscles. In this way, accelerated and decelerated motion becomes possible. To achieve this, the number of force generating cross-bridges is varied by a change in cross-sectional area, that is, by altering the number of fibers recruited for contraction. Thereby the locomotion at high efficiency or maximal power output can be controlled by will. Also, isometric contractions are indispensible for coordinated actions. They are produced by reducing the cross-sectional area to such an extent that a load dependent uncoupling is initiated to stop fiber shortening. 

In many species nervous control of muscles is not a capability which is present from birth on. To reach a certain level of adroitness an individual has to learn—;often during a long lasting phase of exercise—;to control muscle action by coordination. 

## Appendix

### Negative Resistances in Simple Electric Circuits

Reactions occurring in a common car battery can be considered as coupled. A redox reaction is started by introducing a catalyst (the electrodes), which couples the affinity *A_R_* of the redox reaction to the formation of an electrical potential difference Δ*ϕ* at the electrodes. Under open circuit conditions the reaction proceeds rapidly to equilibrium, at which *A_R_* + Δ*ϕ* = 0. Taking A_R_ as the positive input force, then Δ*ϕ* must be negative. *A_R_* can be expressed in electrical units using *E** =* *A_R_/zF* (*E* = electromotive force in Volt V, *z* = charge number, *F* = Faraday constant in Coulombs/Volt, Δ*ϕ* in V). The coupled flow of charges (electrical current in ampere A) is then given by:

(A1)

*E* and Δ*ϕ* correspond to the input force *A_2_* and output force *A_1_*, respectively. *L_c_* is the coupling conductance, and *R_i_* = 1/*L_c_* represents the inner resistance of the battery (*R’s* are given in Ω, *L’s* in 1/Ω). The partial conductances *L_c1_* and *L_c2_* (see equations 2e and 2f) are given by:

, and (A2)

In a simple electric circuit consisting of a battery (*E* = 12 V), an inner resistance *R_i_* (0.2 Ω) and an outer resistance *R_e_* (0.4 Ω), the current *I* is given by *E* - *I*(*R_i_* + *R_e_*) = 0, yielding:

, and from (A1), .

The outgoing voltage delivered to the outer circuit is then given by *U_e_* = −Δ*ϕ* = +8 V.

*L_c1_* and *L_c2_* are given by –5/2 and 5/3 Ω^−1^, respectively, or *R_i1_* = −0.4, and *R_i2_* = 0.6 Ω, which fulfils *R_i1_* + *R_i2_* = *R_i_* = 0.2 Ω. Setting *R_e_* = 4.0 Ω yields *R_i1_* = −4.0, *R_i2_* = 4.2 Ω, and again *R_i_* = 0.2 Ω. Moreover, in the electric circuit the overall resistance *R_i1_* + *R_e_* vanishes. It should be noticed that partial resistances are not constant, although *R_i_* is a constant. They depend both on *R_e_* and *L_e_*, respectively.

The total dissipation function of reactions in the battery and of the outer circuit is given by:

*Ф_circ_* = *Ф_1_* + *Ф_2_* + *Ф_e_* = *I*(*Δϕ* + *E* + *U*), *Ф_circ_* = *I* × *E*(240 J/s or 0.24 kW), or (A3a)

*Ф_circ_* = *L_c2_* × *E*^2^ (1/0.6×144 = 0.24 kW) (A3b)

This latter result means that the total entropy production of the circuit is given by that of the redox reaction. This is also valid with respect to heat production.

Power output as a function of *R_e_* is given by:

(A4a)

, or

Maximal power output is reached at *R_e_* = *R_i_* = 0.2 Ω, *p_out_^max^* = 0.18 kW. Because *P_out_* can also be expressed as *P_out_* = -*L_c1_* × *Δϕ*^2^, this yields with *L_e_* = 1/0.2 1/Ω, *p_out_^max^* = −(−5.0)( −6)^2^ = 0.18 kW.

When a second battery is added to the circuit in such a way that *E^II^* is directed against *E^I^* (*E^II^* = −10.9 V, *R_i_^II^* = 0.5 Ω), Δ*ϕ*^II^ is now positive and *E^II^* negative. The outer resistance *R_e_* stands for the resistance of the wires connecting both batteries. In this constellation, −Δ*ϕ*^I^ is no longer equal to *U_e_*. Now − (Δ*ϕ*^I^+ *U_e_*) = −Δ*ϕ*^Ie^ = Δ*ϕ*^II^ is valid. Consequently, *R_e_* also has to be added to *R_i_^I^* yielding *R_i_^Ie^* = *R_i_^I^*+ *R_e_*.

(A4b)

from:

, and (A4c)

*Δ ϕ^Ie^* = −11.4 V, and Δ*ϕ*^II^ = 11.4 V is obtained.

Partial conductances are given by:

(A4d)

Again, the overall resistance of the electric path in the circuit is zero. The total resistance *R_i_^I^* + *R_e_* + *R_i_^II^* = 1.1 Ω, therefore, is also given by *R_i2_^I^* + *R_i1_^II^* = 12 + (–10.9) = 1.1 Ω.

### ATP, ADP, and P_i_ Species as Functions of [H^+^] and [Mg^2+^]

ATP species, including MgATP^2−^, are calculated according to the methods of Alberty [20]. When respective constants are known, which are dependent on temperature and ionic strength, so-called polynomials can be formulated, from which several parameters like species concentration, *K*'(biochemical equilibrium constant), or [H^+^] and [Mg^2+^] binding can be taken.

ATP splitting by myosin ATPase is formulated here for the species MgATP^2−^, MgADP^−^, and H_2_PO_4_^−^, to make the reactions of the cross-bridge cycle directly dependent on these compounds. The reaction in chemical notation form is given by:

The equilibrium constant for the above reaction is given by:

(A5a)

(A5b)

(A5c)

K^ref1^ (= 6.267 × 10^5^), K3at, K3ad, *P_ATP4−_*, and *P_ADP3−_* were taken from [1]. At given [H^+^] and [Mg^2+^] values, the corresponding *K*'(= 4.9687 × 10^5^, pH = 7.1, [Mg^2+^] = 800 µM) is identical to formulations with other reference constants.

### [H^+^] and [Mg^2+^] Buffering

[H^+^] buffering of SMFs is treated here analogously to VMs (see [1]). It is given by:

(A6a)

At very high ATP consumption, which may not be matched by ATP production differences in proton binding between substrates and products of reactions like ATP splitting, CK and AK reactions may also contribute to pH changes. According to [20], such changes for [H^+^] and [Mg^2+^], respectively, are given by:

, and (A6b)

, and (A6c)

as a result,

(A6d)

is identical to *κ_H_^BU^* if binding sites contain only a single site with only one proton dissociation constant.

For [Mg^2+^] buffering, it is suggested that during short time intervals Mg^2+^ transport reactions across membranes can be neglected. Only intrinsic binding sites including [ATP] are present and, as with [H^+^] changes, [Mg^2+^] changes induced by ATP splitting, the CK reaction, and the AK reaction have been addressed. [Mg^2+^] buffering can be expressed as:

(A7a)

(A7b)

(A7c)

In addition, Mg^2+^ binding depends on [H^+^]. A decrease of pH can liberate magnesium ions from intrinsic binding sites and from the predominant ATP species MgATP^2−^. The H^+^ and Mg^2+^ dissociation constants of both binding sites are set to the values of a simplified *P_ATP4−_*. The total concentration of Mg^2+^ binding sites, , is adjusted to 9.0 mM plus a variable [ATP]. The change of [Mg^2+^] is given then by:

(A7d)

In simulations, instead of complete d[H^+^]/dt, only those fluxes producing or consuming protons are considered, because changes of [H^+^] depend mainly on these fluxes (see Figure 5A).

[Mg^2+^] is introduced as a variable only in those simulations that deal with muscular fatigue. Because changes of [Mg^2+^] depend mainly on acidification, and pH does not change markedly even under conditions of high power output, this variable is set constant to 800 µM for all other simulations.

In the above equations, methods of calculus are used so formulas can be held compact. In simulations, however, these equations must be incorporated in an explicit form, which often results in very voluminous expressions.

### Simulation of Glycogenolysis and Glycolysis

Most flux equations of glycogenolysis are congruent with those of a simulation of glycolysis given in [1]; they are taken over from that article. Glucose-6-phosphate (G6P) formation by hexokinase (HK) and glycogen phosphorylase is now included. The new flux equations used here are as follows.

Flux through glycogen phosphorylase:

(A8)

*L_Phosph_^max^* = 4×10^−3^ (µM/ms)×(mol/J), *K_M_^Phosph^* = 2.0 µM, K'_Phosph_ = 0.286;

glucose – 6 –phosphate isomerase,

(A9)

*L_GPI_^max^* = 2×10^−2^ (µM/ms)×(mol/J *K_M_^GPI^*), = 300 µM, K'_GPI_ = 0.276;

lactate dehydrogenase,

(A10)

*L_LDH_^max^* = 2.4×10^−2^ (µM/ms)×(mol/J), *K_M_^ldh^* = 50 µM, K'_LDH_ = 2.497×10^4^;

lactate/proton cotransport,

(A11)

*G_Lac_^max^* = 2.866× 10^8^ pS (pico Siemens = 10^−8 ^Ω^−1^), *K_M_^Lac^* = 17 mM;

Na^+^/H^+^ exchange,

(A12)

*G_NaH_^max^* = 10^5^ pS, H_05_ = 0.1 µM, S_[H_^+^_]_ = 0.004 µM;

anion exchange reaction,

(A13)

*G_AnEx_^max^* = 10^4^ pS, H_05_ = 0.05 µM, S_05_ = 0.008 µM, *K_M_^anex^* = 13.0 mM.

The energising flux of the cross-bridge cycle is given by:

(A14)

*L_En_^max^* = 6.138×10^−2^ (µM/ms)×(mol/J), *f_corr_* = ([CB_t_]−[CB_0_])/([CB_t_]−[CB]), [CB_t_] = 656 µM, [CB_0_] = 492 µM, *ε* = 24.0, A_L05_ = 3.0×10^4^ (J/mol), S_L_ = 2.0×10^4^ (J/mol), , *A_En_* = −5.866729×10^4^, .

The factor [CB]/[CB_0_] corrects *L_En_^max^* for changes of [CB]; the second factor is introduced to damp changes of [CB]. *K_B_^ref^* is not constant, but depends on [Ca^2+^].

The stroke generating fluxes are given by:

(A15a)

(A15b)

Both fluxes are identical as long as uncoupling is absent. The first factor corrects *L_Str_^max^* = 4.6 × 10^−4^ (µM/ms)×(mol/J) for changes of [CB_t_] – [CB]. The second factor introduces [Ca^2+^] dependence of *L_Str_*. The third factor is responsible for the hyperbolic character of the flux equation at constant [Ca^2+^] with *K_I_^CB^* = −1.8 × 10^4^ J/mol, which represents the inhibition constant, *K_R_^ref^* = 1.310889 × 10^−4^. λ values are not independent; this interdependency is given in Results. Uncoupling is formulated to occur in two steps, expressed by *λ_Str1_^P^* = 0.15 and *λ_Str2_^P^* = 0.85.

SIMGLYgen (A16)

.

.

.

.

.

.

.

.

.

.

.

.

.

The above set of differential equations without a variable [Mg^2+^] ([Mg^2+^] = 0.8 mM = const.) is used to calculate the various points of figures (Figure 1B, Figure 2, Figure 3, and Figure 4) for a given [Ca^2+^] and various loads. As already mentioned, [Mg^2+^] is introduced as a variable only for conditions of very high power output leading to fatigue. From the output of the simulation many more variables, as shown here, can be obtained as functions of time, which may often be helpful in understanding underlying mechanisms.
